# Green microbes: Potential solutions for key sustainable development goals

**DOI:** 10.1111/1751-7915.14546

**Published:** 2024-08-10

**Authors:** Sandra Diaz‐Troya, María José Huertas

**Affiliations:** ^1^ Departamento de Bioquímica Vegetal y Biología Molecular, Facultad de Biología Universidad de Sevilla Sevilla Spain; ^2^ Instituto de Bioquímica Vegetal y Fotosíntesis, Universidad de Sevilla, Consejo Superior de Investigaciones Científicas Sevilla Spain

## Abstract

The latest assessment of progress towards the Sustainable Development Goals (SDGs) has identified major obstacles, such as climate change, global instability and pandemics, which threaten efforts to achieve the SDGs even by 2050. Urgent action is needed, particularly to reduce poverty, hunger and climate change. In this context, microalgae are emerging as a promising solution, particularly in the context of food security and environmental sustainability. As versatile organisms, microalgae offer nutritional benefits such as high‐quality proteins and essential fatty acids, and can be cultivated in non‐arable areas, reducing competition for resources and improving the sustainability of food systems. The role of microalgae also includes other applications in aquaculture, where they serve as sustainable alternatives to animal feed, and in agriculture, where they act as biofertilizers and biostimulants. These microorganisms also play a key role in interventions on degraded land, stabilizing soils, improving hydrological function and increasing nutrient and carbon availability. Microalgae therefore support several SDGs by promoting sustainable agricultural practices and contributing to land restoration and carbon sequestration efforts. The integration of microalgae in these areas is essential to mitigate environmental impacts and improve global food security, highlighting the need for increased research and development, as well as public and political support, to exploit their full potential to advance the SDGs.

## Graphical abstract

Microalgae, with their photosynthetic and biotechnological capabilities, have significant potential to address the Sustainable Development Goals (SDGs). These microorganisms can help fight hunger, improve agricultural sustainability and mitigate environmental impacts, offering a promising pathway towards resilient and sustainable practices.

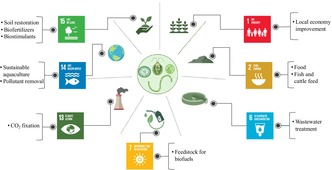

## CURRENT STATUS OF SDG ACHIEVEMENT

Last year, a group of 104 scientific experts from various fields assessed progress towards achieving the Sustainable Development Goals (SDGs) (Sustainable Development Goals Report 2023: Special Edition, Towards a rescue plan for people and planet, 2023). Although this report is produced every 4 years, the mid‐term review of the 2030 Agenda is an important opportunity to assess the results achieved to date. Unfortunately, the conclusions are not encouraging. The main obstacles to progress towards the SDGs are the impact of the climate crisis, wars, the weakness of the global economy and the drastic impact of pandemics. Although the lack of progress is universal across the SDGs, it is the world's poorest and most vulnerable who are bearing the brunt of these unprecedented global challenges. The report concludes that the world is not on track to achieve the Sustainable Development Goals. More worryingly, at the current rate of progress, these goals seem unattainable even if they are delayed until 2050 (Soergel et al., 2021). There are areas where urgent action is needed to rescue the SDGs and achieve meaningful progress for people and the planet. These include addressing and finding solutions to the increasing number of people who will remain in extreme poverty (No poverty and Zero hunger), and the rise in global temperature (which has already reached 1.1°C above preindustrial levels and is projected to reach or exceed 1.5°C by 2035).

Therefore, it is imperative to increase efforts to achieve these goals, with contributions from various sectors of society, including science and biotechnology. In this sense, the contribution of microbial biotechnology to the SDGs is key and has already been addressed in a general way in another issue of the MBT (Soberón‐Chávez, 2023). In this article, we will focus on the most promising aspects of a specific group of microorganisms: green microbes or microalgae, which are candidates to contribute to solving some problems of great importance for human health and the environment that affect developing countries.

## MICROALGAE AND CYANOBACTERIA

In biotechnology, the term microalgae is commonly used to refer to both cyanobacteria and eukaryotic microalgae. These photosynthetic microorganisms are found in a wide range of habitats, from deserts to glaciers, and are particularly important in aquatic ecosystems. They have been estimated to be responsible for around 50% of the world's net primary production (Field et al., 1998).

Microalgae offer several advantages for use in biotechnological processes. They have simple nutritional requirements and can be grown in wastewater, reducing competition with traditional crops for soil and water resources (Winckelmann et al., 2015). In addition, their photosynthetic capacity allows them to couple CO_2_ uptake and fixation with organic compounds production, contributing to the environmental sustainability of the processes. It should be noted that some cyanobacteria are also able to fix N_2_. Recently, this function has also been observed in the eukaryotic microalga *Braarudosphaera bigelowii*, which carries a unicellular cyanobacterium as a new type of bona fide organelle, the nitroplast (Coale et al., 2024). Microalgae show great metabolic plasticity, biodiversity and tolerance to extreme environments (Metting, 1996), which makes them a potential source of new compounds. These compounds range from molecules with therapeutic activity to toxins harmful to human health and the environment (Baunach et al., 2023). Finally, genetic manipulation tools exist for these microorganisms, in particular for some model cyanobacteria such as *Synechocystis* or *Anabaena*. Although these tools are not as well developed as for other model organisms such as *E. coli* or *S. cerevisiae*, significant advances have been made in both the techniques available (Fayyaz et al., 2020) and the organisms that can be manipulated (Jester et al., 2022). Therefore, the use of microalgae and cyanobacteria that fix CO_2_ and from which biomass or by‐products can be obtained would close the loop in a circular economy strategy, in line with the principles defended in the Farm to Fork Strategy declared by the European Commission, making food systems fair, healthy and environmentally friendly (Björkbom, 2023).

**FIGURE 1:**
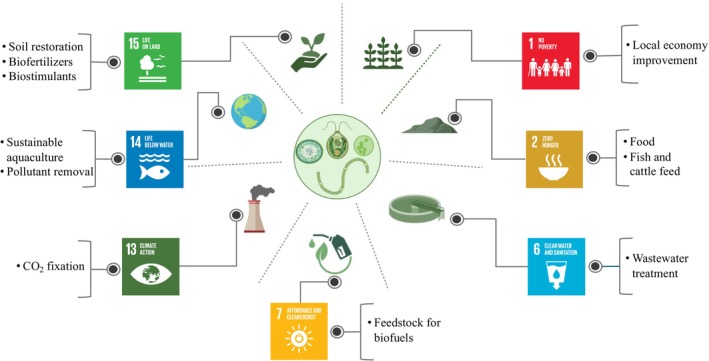
Conceptual diagram of the contribution of microalgae to the achievement of the Sustainable Development Goals.

Microalgae can directly or indirectly contribute to numerous SDGs. In this ‘Burning Questions’, we will focus primarily on how microalgae can advance three specific SDGs, ‘Zero hunger’, ‘Climate action’ and ‘Life on land’ (Figure 1) while also briefly exploring their influence on other SDGs.

## HOW CAN MICROALGAE CONTRIBUTE TO ACHIEVING THE ‘ZERO HUNGER’ SDG2?

With the global population exceeding 8 billion and projected to continue growing, particularly in Africa and Asia, it is imperative to take action to guarantee access to nutritious food for this growing population (Feeding the future global population, 2024). Furthermore, when considering indicators that cover various aspects of food systems, such as nutrition and health, natural resources, equity, governance and resilience, no single region or income group has a desirable situation on all indicators (Schneider et al., 2023). Therefore, it is essential to address the social and environmental impacts of food production and consumption and consider alternatives to healthier and more sustainable diets.

The utilization of microalgae in human food may seem like a recent trend; however, historical evidence indicates that they have been consumed for centuries. The chronicles of Bernal Diaz, who accompanied Hernán Cortés on his arrival in Tenochtitlan, describe the consumption of tecuitlatl (Spirulina or *Arthrospira*) by the Aztecs of Lake Texcoco (Farrar, 1966). Spirulina is a promising microorganism for sustainable food production. Its rapid growth, high nutritional value and low environmental impact make it an excellent candidate for addressing global food security and sustainability challenges (Choi et al., 2022). Several species of the genus *Nostoc*, as well as *Spirogyra* and *Oedogonium*, are currently consumed by local populations in regions such as Africa (Lake Chad), South America and particularly Asia (Gantar & Svirčev, 2008). Although the nutritional composition varies between the different species of microalgae, and even within the same species depending on the environmental and cultivation conditions, they have a very interesting nutritional profile. They represent a very valuable potential source of protein in terms of both quantity and quality. Therefore, species such as *Arthrospira platensis* can reach a protein content of 70% of their dry weight (Lafarga et al., 2020). Furthermore, unlike most vegetable proteins, most species of microalgae currently considered safe for human consumption contain all essential amino acids (Torres‐Tiji et al., 2020). We can also find species with the capacity to accumulate very high levels of lipids, such as *Auxenochlorella protothecoides*, which can accumulate up to 70% of its dry biomass as lipids (Heredia‐Arroyo et al., 2010; Rismani‐Yazdi et al., 2015). Again, the type of fatty acids that some microalgae accumulate is also interesting. Therefore, we can find microalgae with essential fatty acids or with a high content of long‐chain polyunsaturated fatty acids (PUFAs) (Matos et al., 2016), with beneficial health effects (Khan et al., 2023). PUFAs are incorporated into the diet through the consumption of cold‐water fish, which in turn, obtain them from the consumption of microalgae. Therefore, the incorporation of microalgae consumption into the diet can lead to a significant improvement in nutritional quality, especially in plant‐based diets (Lupette & Benning, 2020).

Despite their nutritional benefits and the efforts of organizations such as the FAO, microalgae have not been widely adopted as a food source (Cai et al., 2021; Habib et al., 2008). This is due, among other factors, to consumers' reluctance to change their eating habits and palatability issues of microalgae. Improving the sensory qualities of microalgae is challenging due to the complex range of molecules that contribute to aroma and taste (Urlass et al., 2023). Despite this, there is an increasing market for microalgae as a food ingredient (Lafarga, 2019). Strategies to increase microalga consumption in society would involve raising awareness among consumers of their nutritional benefits and low environmental impact, as well as improving their taste through modifications in cultivation methods, post‐harvest processing, or cooking. Genetic modification could also be used to produce molecules that affect taste, such as sweeteners or volatile flavouring compounds like limonene or 1,8‐cineole (Chenebault et al., 2023; Sakamaki et al., 2023) or to eliminate them, such as chlorophyll‐deficient mutants of *Chlorella vulgaris* (Schüler et al., 2020).

In addition to its use as human food, the utilization of microalgae in animal feed, particularly in aquaculture, is also noteworthy. In a world where the demand for animal protein is increasing and fish consumption has led to overexploitation of fish stocks, aquaculture is emerging as a necessary alternative (Naylor et al., 2021). However, this approach also places significant pressure on aquatic resources due to the extensive use of fishmeal and fish oil as the primary sources of protein and fat in aquaculture feeds. SDG 14 ‘Life below water’ aims to promote sustainable use of marine resources. To effectively address this challenge and promote environmental sustainability in the aquaculture sector (included in Task 14.7), it is crucial to explore new feed alternatives for use in aquaculture production. Microalgae are widely used as both direct and indirect feed in aquaculture. Live microalgae are commonly used as a direct feed for larvae and molluscs. Microalgae can also serve as feed for zooplankton, such as rotifers, Artemia and copepods. These zooplankton are then used to feed fish and molluscs at later stages of development (Ma & Hu, 2024).

Microalgae are a promising alternative to fishmeal and fish oil in aquaculture feed formulation due to their high protein content, optimal nutritional profile and functional compounds such as PUFAs, pigments and vitamins. *Spirulina* and *Chlorella* are among the most used microalgae species as protein sources to replace fishmeal (Alagawany et al., 2021). The percentage of substitution varies depending on the species of fish, the stage of growth and the type of microalgae used. Microalgae, such as *Schizochytrium* or *Crypthecodinium cohnii*, have also been investigated as total or partial substitutes for fish oil in formulated feed for species including Atlantic salmon, rainbow trout or Pacific white shrimp (Betiku et al., 2016; Sprague et al., 2015; Wang et al., 2017). These eukaryotic microalgae have a high content of PUFAs, making them a superior alternative to vegetable oils as substitutes for fish oil in fish feed formulation.

In this case, one of the biggest problems for a wider implementation of the use of microalgae in aquaculture is the economic factor. Despite the implementation of cost‐reducing improvements in microalgae production systems, the cost of producing microalgae as aquaculture feed ingredients remains considerably higher than that of fishmeal and fish oil. Therefore, further development is necessary in production systems to make the process economically viable (Nagappan et al., 2012).

Another important aspect of SDG 2 ‘Zero hunger’ is to promote sustainable agriculture. It is crucial to increase productivity, especially among small‐scale producers, in an environmentally sustainable way and with resources that enable them to adapt to climate change, droughts and other environmental disasters (targets 2.3 and 2.4). However, the techniques employed in traditional intensive agriculture to increase productivity often led to environmental degradation. One of the main issues is the use of pesticides and fertilizers (mainly rich in N and P), which can result in chemical contamination of soil and water, as well as eutrophication of water bodies through soil leaching, which negatively impact SDG 6 (Clean water and sanitation), SDG 14 (Life below water) and SDG 15 (Life on land). An approach to developing more sustainable agricultural practices involves the use of bioproducts such as biofertilizers, biostimulants and biopesticides. Briefly, the main differences among them are that biofertilizers are biological products that contain microorganisms or compounds derived from them, enhancing soil fertility and plant growth by increasing nutrient availability, while biostimulants modulate plant metabolism, promoting growth and tolerance to environmental stress, but do not directly supply nutrients to the plant. Biopesticides, on the other hand, control pathogenic organisms through biological means (Ferreira et al., 2023).

Microalgae are currently attracting a great deal of attention in sustainable agriculture for their use as bioproducts. Similar to their use in food, cyanobacteria have traditionally been used as biofertilizers in agriculture as they play a crucial role in biological nitrogen fixation, offering a sustainable and environmentally friendly solution to the planet's nitrogen dilemma. Their ability to convert atmospheric nitrogen, either independently or in consortium with other microorganisms, into a usable form without relying on fossil fuels makes them a key component in the shift towards more sustainable agricultural practices, thus reducing dependence on conventional fertilizers (Matassa et al., 2023). Nitrogen‐fixing cyanobacteria have been used as biofertilizers in rice cultivation in regions of China and Vietnam (Yao et al., 2018). The fixing capacities of indigenous cyanobacteria in agricultural soils are also being harnessed for use as biofertilizers in cotton soils, where they have been shown to be effective in improving cotton growth. (Jiménez‐Ríos et al., 2024).As technology advances, it is becoming increasingly feasible for cyanobacteria to replace traditional nitrogen fertilizers and contribute to global food security and environmental sustainability.

In addition, both cyanobacteria and eukaryotic microalgae have been found to improve the bioavailability of other macronutrients, such as P, and micronutrients, such as Cu, Fe, Mn and Zn, resulting in improved plant growth. Several species of microalgae belonging to the genera *Nostoc*, *Anabaena* and *Chlorella* have been used for these purposes (Ferreira et al., 2023).

In addition to their role as biofertilizers, microalgae exhibit remarkable potential as biostimulants, influencing various aspects of plant physiology to improve crop productivity. They can affect root development, which subsequently impacts overall plant growth, flowering, fruit production, germination, and resistance to biotic and abiotic stresses such as saline stress. The diverse metabolites produced by the microalgae responsible for these effects include phytohormones such as auxins and gibberellins, amino acids, polysaccharides and antioxidants (Kapoore et al., 2021).

## HOW CAN MICROALGAE CONTRIBUTE TO ACHIEVE THE ‘CLIMATE ACTION’ SDG 13?

Carbon dioxide levels in the atmosphere are expected to increase dramatically in the coming years. In this scenario, the concept of CO_2_ mitigation and sequestration using photosynthetic microorganisms is of great interest (Mehta et al., 2019). The use of cyanobacterial species has been identified as one of the most promising methods for CO_2_ sequestration and as a feedstock to produce biofuels to replace fossil fuels (SDGs 7, 13). Cyanobacteria are an ideal starting point for the development of artificial systems using synthetic biology to convert atmospheric carbon into useful products. One example is the design of metabolic pathways to produce 2,3‐butanediol (Gonzales et al., 2023; McEwen et al., 2016), isopropanol (Hirokawa et al., 2017, 2020) or sucrose (Santos‐Merino et al., 2023; Yun et al., 2024).

Methods to reduce carbon dioxide to C1 molecules have also attracted considerable interest. Cyanobacteria have the potential to work synergistically with other microorganisms to enhance the conversion of CO_2_ and other C1 compounds into valuable chemicals (García & Galán, 2022). For example, the photomixotrophic metabolism of cyanobacteria can be combined with methanotrophic bacteria to convert CO_2_ and CH_4_ into more complex and valuable compounds (Kanno et al., 2017; Singh et al., 2018). This integrated approach can lead to higher yields and more efficient bioprocesses. In addition, the creation of artificial bacterial consortia, including cyanobacteria, is a promising strategy to improve the efficiency of C1 compound conversion (Santos‐Merino et al., 2023). These consortia can be engineered to optimize the metabolic pathways of each microorganism, thereby improving overall productivity and sustainability.

In conclusion, cyanobacteria are essential for the capture and conversion of greenhouse gases. Their photosynthetic capacity and genetic engineering potential make them ideal candidates for the development of sustainable and efficient bioprocesses. The integration of cyanobacteria into various biotechnological systems is a promising approach to address the challenges of climate change.

## HOW CAN MICROALGAE CONTRIBUTE TO ACHIEVE THE ‘LIFE ON LAND’ SDG 15?

Arid and semi‐arid environments cover 41% of the world's land area and support more than 38% of the world population. Climate change, human activities and other factors are affecting about 25% of the world's land, and more than 40% of the Earth's land is at risk of desertification (Ramakrishnan et al., 2023). In response to this urgent challenge, target 15.3 of the SDGs sets the goal of reducing desertification, restoring degraded land and soil, including areas affected by desertification worldwide by 2030. This target underlines the need not only to stop the spread of desertification but also to reverse the damage already done to our planet's vital ecosystems, ensuring the resilience and sustenance of the populations who depend the most on these lands. The formation of cyanocrusts in these arid soils is crucial to stabilizing soil structure, minimizing erosion, and promoting plant growth as biofertilizers and biostimulants, highlighting the importance of their inoculation in restoring dry and degraded soils. Cyanocrusts play an essential role in soil stabilization by forming an organic sedimentary conglomerate within the soil that favours the development of microbial populations. Its initial colonization by non‐heterocytic motile filamentous cyanobacteria creates a physical structure that traps mineral particles with exopolysaccharides, resulting in a stabilized soil environment. This facilitates the colonization of more complex communities of non‐motile cyanobacteria and other microorganisms, promoting the formation of microenvironments and supporting important biological activities (Garcia‐Pichel, 2023). Cyanocrusts stabilize soils, improve hydrological function, and increase nutrient and carbon availability. Understanding the composition of the microbial communities that form the cyanocrusts and the methodology for their restoration, when lost from ecosystems, is key to the successful restoration of ecosystems (Antoninka et al., 2020). Nelson and colleagues have analysed the spatial organization of soil cyanobacteria, particularly *Microcoleus vaginatus*, showing how cyanobacteria form bundles that create a mutually beneficial environment for other microbes, and how signals such as GABA facilitate this assembly. These studies demonstrate that specific molecular interactions can shape microbial communities and influence soil health and productivity. This advances our understanding of soil microbiomes, with implications for ecology and agriculture (Nelson et al., 2024).

Furthermore, cyanocrusts help reduce atmospheric CO_2_ through photosynthesis in the soil, using both atmospheric and soil carbon dioxide. The use of CO_2_ demonstrates the crucial role of cyanobacteria in the carbon cycle of arid ecosystems and provides a natural method of CO_2_ removal and soil stabilization.

Saline soils are a global problem that affects agricultural productivity and environmental health. These soils are characterized by a high salt content, which can be detrimental to plant growth, resulting in reduced agricultural yields. Remediation strategies are needed to improve soil quality and reclaim land for agricultural use. One of the proposed methods is the potential use of microalgae, which could play an important role in soil health and sustainability. Microalgae can remove salt from the soil by adsorption, controlled by the cell wall, and excretion of extracellular polymeric substances (EPS). Recently, Pei and colleagues demonstrated the creation of microalgae ecofarms on site. Microalgae grown at these sites would be cultivated with nearby brackish water as a nutrient source and used for soil remediation and biomass production (Pei & Yu, 2023) Salt‐tolerant microalgae species could accelerate the remediation process. Therefore, the search for and characterization of microalgal species with high salt tolerance and the identification of the mechanisms involved in this adaptation are of great interest. Freyra and coworkers show that microalgae adapt to different salinities by using specific metabolic pathways, transcription factors and protein kinases. In particular, Na+/H+ antiporters and Na+/Pi symporters were overexpressed at lower salinities, with a strategy of using K+ channel complexes at higher salinities. This provides valuable insights on the genomic basis of salinity tolerance, and the search for and characterization of microalgal species with high salt tolerance and the identification of the mechanisms involved in this adaptation are of great interest. This research adds valuable knowledge to the genomic basis for salinity tolerance (Freyria et al., 2022). Similarly, another interesting approach is the design of strains with improved salt‐tolerance characteristics, such as the synthesis of compatible solutes (Dong et al., 2023).

## CONCLUSIONS AND PROMISING FUTURE

Exploring the diverse roles of microalgae and cyanobacteria in support of SDGs highlights their great potential. Although they face significant challenges in their large‐scale application and cultivation, their innate capabilities position them as crucial allies in the quest for a more sustainable future. Furthermore, it is important to find solutions to problems, especially in developing countries where technology does not reach them in the same way. In this sense, Jester et al. have published a successful example of how the potential of these microorganisms can be applied to problems in developing countries with fewer resources (Jester et al., 2022). They present significant advances in the genetic engineering of *Arthrospira platensis* (spirulina) for the production and oral administration of therapeutic proteins. Despite limitations due to the lack of genetic tools for spirulina, this study presents methods for stable, high‐level expression of bioactive proteins in spirulina, including large‐scale culture techniques and downstream processing methods. Targeted integration of exogenous genes into the spirulina chromosome allows the biofabrication of biopharmaceuticals that represent a substantial part of the total biomass. These biopharmaceuticals do not require purification prior to oral administration, are stable without refrigeration and are protected during gastrointestinal transit when encapsulated in dried spirulina. Oral administration of spirulina‐expressed antibodies against campylobacteriosis, a major cause of infant mortality in developing countries, has shown efficacy in animal models and safety in a phase 1 clinical trial. This makes spirulina a promising biopharmaceutical for the production of orally administered therapeutic proteins with a high impact on human health in developing countries.

The ability of microalgae to adapt and proliferate in a wide variety of environments, their efficiency in photosynthesis, and their utility in biotechnological applications offer promising opportunities to address some of the most pressing problems of the world. By promoting advances in microbial biotechnology, we can harness the untapped potential of these microorganisms in a wide range of sectors, including food production and environmental protection. Promising applications of microalgae include reducing hunger, improving nutrition and promoting sustainable agriculture and aquaculture. As we face a future of increasing environmental and societal challenges, the role of these green microorganisms in achieving a land degradation neutral world and supporting a circular economy will undoubtedly be indispensable.

Through strategic investment in research and development, coupled with public awareness and political support, the integration of these organisms into global sustainability efforts can be optimized. These efforts are not only in line with the SDGs but also represent a paradigm change towards resilient and sustainable practices that could transform our relationship with the environment and its invaluable biological resources.

## AUTHOR CONTRIBUTIONS

**Sandra Diaz‐Troya:** Writing – review and editing; supervision. **María José Huertas:** Conceptualization; writing – review and editing; supervision.

## CONFLICT OF INTEREST STATEMENT

The authors declare no conflict of interests.

## Data Availability

Data sharing not applicable to this article as no datasets were generated or analysed during the current study.
